# Intense Endurance Exercise: A Potential Risk Factor in the Development of Heart Disease

**DOI:** 10.7759/cureus.12608

**Published:** 2021-01-10

**Authors:** Jocelynn Rajanayagam, Mohammed Alsabri

**Affiliations:** 1 Medicine, Saba University School of Medicine, Ontario, CAN; 2 Paediatrics, Brookdale University Hospital Medical Center, Brooklyn, USA

**Keywords:** cardiac changes, endurance exercise, athletes

## Abstract

Exercise is known to improve cardiac health; however, the upper limit of exercise is not as clear. High-intensity endurance exercise and its association with cardiac dysfunction are becoming more important as more people in the USA participate in endurance activity. Intensive endurance exercise is associated with heart disease.

Peer-reviewed primary literature related to endurance exercise, cardiac remodelling, myocardial fibrosis, and arrhythmia was searched and selected from the PubMed Database to be reviewed. Increased cardiac remodelling, myocardial fibrosis, and arrhythmias were associated with increased endurance exercise in most instances. Clinically significant dysfunction from endurance exercise-induced cardiac remodelling and myocardial fibrosis is not certain. However, increased incidence of sinus node disease (SND) and atrial flutter in endurance athletes, even after deconditioning, seems to be most associated with endurance exercise.

Cardiac remodelling, fibrosis, and arrhythmia were associated with cardiac dysfunction. All review articles in this study were limited by a low sample size since it was difficult to obtain a full record of previous endurance activity. The reversibility of cardiac changes needs to be further investigated and can be applied in the differential diagnosis of other cardiomyopathies. The interaction between performance-enhancing drugs and endurance exercise needs to be further studied to determine its action on cardiac health. Findings from these studies could contribute to exercise recommendations.

## Introduction and background

Heart disease incidence and risk factors

In 2018, heart disease (HD) was the leading cause of death worldwide and accounts for 15.2 million deaths [1]. HD includes a variety of pathologies of the heart; such as myocardial fibrosis, cardiomyopathies, systolic and diastolic dysfunctions, and arrhythmias [2 ]. All of these conditions could lead to further cardiovascular remodelling and death. There are modifiable and unmodifiable risk factors associated with HD. The non-modifiable factors linked to increased risk for developing HD are older age, male gender, African or Asian ethnicity, and family history of HD. Some of the modifiable risk factors that increase the risk for HD are tobacco use, obesity, diets high in sodium and fat, and poorly controlled diabetes and hypertension [2].

Exercise recommendations and endurance exercise in the USA

Fortunately, there are known solutions that can counteract the modifiable risk factors for developing HD. Long-term moderate exercise is one of the most important interventions that has been proven to reduce body weight, normalize insulin sensitivity, reduce cholesterol, and normalize blood pressure [3]. The exercise guidelines for adults are 2.5 hours to 5 hours a week of moderate exercise or 1.25 hours to 2.5 hours of vigorous-intensity, aerobic exercise once a week. This is also recommended to be in combination with weight training of all muscle groups two times a week [3]. Following these recommendations ultimately has a lasting impact on reducing the risk of developing HD and mitigating the effects of existing HD [4]. Exercise unequivocally results in many physical and psychological health benefits; however, the upper limit of exercise and its implications on cardiac health are not as clear. Athletic hearts structurally and physiologically adapt towards the body’s increased oxygen demand during extensive exercise [4]. For instance, hearts of athletes are larger in mass, have an increased right and left ventricular end-diastolic volume (EDV) and increased contractility, resulting in increased lung perfusion and cardiac output (CO) [5]. However, it has also been suggested that individuals who participate in long-term and intense endurance exercise have a higher prevalence of cardiovascular complications than their sedentary counterparts [6]. The association between endurance exercise and HD is becoming more important since self-trained individuals within the general public are participating in highly intensive endurance activities that sometimes require vigorous exercise well beyond the recommendations by the CDC. In the USA, the number of individuals who participated in triathlons increased from 0.92 million in 2006 to 4.08 million in 2017 [7]. This does not include other forms of extensive endurance exercise such as marathons, cycling, and canoeing. While these forms of exercise promote healthier lifestyles, improper modifications in diet along with excessive training may have an association with HD. It has been suggested that intense endurance exercise could lead to increased cardiac remodelling, myocardial fibrosis, and arrhythmias which could ultimately progress to cardiac dysfunction [8]. Therefore, it is important to understand the pathophysiological process of HD induced by endurance exercise in order to recognize the upper limit of exercise within different patient populations. 

Pathophysiology of HD induced by endurance exercise

Long-term exercise induces many different changes in the heart in order to adapt to the increase in oxygen demand of the body. The entire heart increases in mass which leads to increased contractility and EDV; thereby, increasing stroke volume (SV) and lung perfusion [8]. This ultimately allows for more blood to become oxygenated by the lungs and delivered to tissue. Over time, the heart also displays a parasympathetically mediated bradycardia since a higher blood volume is pumped out of the heart for every beat [8]. In a healthy individual, the stroke volume and heart rate reach an equilibrium where the CO is sufficient for the oxygen demand of the body [5]. It has also been observed that the coronary arteries become more dilated in order to meet the increased oxygen demand of the heart itself. These adaptations are referred to as ‘athlete’s heart’. [5] Although an athlete’s heart is not pathological, increased exercise intensity and duration could exacerbate these changes and cause maladaptation. For instance, excessively dilated heart chambers, myocardial fibrosis, and arrhythmias are some of the pathological changes induced by exercise [9]. Dilated ventricles are observed in athlete’s heart; however, when the ventricles are dilated to their maximum capability and are subjected to a high load during exercise, contractile dysfunction may occur due to myocyte injury [10]. Since the right ventricle (RV) normally operates under lower pressures than the left ventricle (LV), the RV has a lower contractile force and is more susceptible to dilation fibers [10]. Due to the lower capability of the RV to withstand high blood load during intense exercise, it becomes maximally dilated sooner than the LV and undergoes myocardial damage. Ventricular dysfunction could also be due to myocardial fibrosis. Intense endurance causes very high flow and pressure within the vessels and chambers which overwhelms the maximally dilated arteries [11]. This induces the release of inflammatory cytokines and accelerates injury within the vessels and the heart itself. Some inflammatory cytokines released during exercise are TNF-alpha, N-terminal pro-brain natriuretic peptide (NT-proBNP), and cardiac troponin T (cTnT) [11]. The heart has a reparative response to the damaged myocytes by inducing fibrosis. The increased collagen deposition in the myocardium has the potential to decrease compliance and contraction of the ventricles [9]. Compromised outflow and increased pressure in the ventricles could lead to atrial enlargement; thereby, inducing atrial fibrillation (AF) [12]. Atrial enlargement (especially seen in the left atria due to high pressure in the LV) causes morphological changes in the conduction pathways within the heart - this is known as arrhythmogenic remodelling [13]. It has also been suggested that the increased vagal tone and bradycardia in athletes could increase the risk of developing AF and sinus node disease (SND) [14].

Hypothesis

Exercise undoubtedly provides many health benefits such as normalizing body weight and insulin sensitivity. These benefits are known to decrease the risk of developing cardiac dysfunction. However, it has also been observed that intense and long-term endurance exercise could have an association with inducing HD. Therefore, the purpose of this literature analysis is to investigate the possible pathological processes that occur during exercise and whether there are other underlying factors that could account for cardiac impairment seen in endurance athletes. It is hypothesized that intense endurance exercise induces cardiac dysfunction through chamber remodelling, fibrosis, and arrhythmia.

## Review

Methods

The articles reviewed in this literature analysis were retrieved from the PubMed database where the most recent search was conducted on April 8, 2019. The keywords used in PubMed were: (“myocardial fibrosis” OR “calcification” OR “ischemia” OR "atrial fibrillation" OR "dilation") AND (“endurance exercise” OR “long-term exercise” OR “endurance athletes” OR "marathon" OR "athletes"). This search generated 792 results.

Inclusion criteria were primary literature that used animal models, randomized control trials with animals, and human prospective/observational cohort or case-control studies. The articles included also investigated the association of exercise-related HD and possible pathophysiological mechanisms that may occur during exercise. The researchers followed ethical procedures and received consent in investigations involving humans.

Exclusion criteria were any form of secondary or tertiary literature; such as meta-analyses and review articles. Control trials involving human subjects, case series, articles that were published before 2000, and non-English literature were excluded.

Ten of the remaining 661 articles were used in this literature analysis. Although the search had articles from 2000 onward, 9 of the 10 selected articles were from 2006 or later. The investigation by Pelliccia et al. [15] was included in this analysis since it was one of the few investigations that directly evaluated the reversibility of cardiac changes in humans.

Results

The literature selected were all primary articles that focused on the association of HD and endurance exercise as well as the pathophysiological mechanisms that may facilitate this disease process. The following section will outline the relevant methods and findings of all 10 articles. The first three articles focus on the effect of endurance exercise on cardiac remodelling such as dilation. The next four articles concentrate on the development of fibrosis caused by endurance exercise. Finally, the last three articles are related to the presence and cause of arrhythmia in endurance athletes.

Cardiac Remodelling and Dysfunction

Apor et al. [6] recruited 22 elite athletes (mainly canoers who competed on the international level) and 21 controls who have never participated in competitive sports age 27 ± 10 and 30 ± 5 years old, respectively. They used a 2D Doppler echocardiogram in order to observe any changes in blood flow through the heart after intense exercise. The stiffness and preload of the heart were evaluated using parameterized diastolic filling (PDF) formalization which served as an indicator for cardiac function. The PDF formalization utilized the information from the Doppler echocardiogram and provided load-independent measurements of diastolic function. The measurements were recorded at rest in both groups in order to determine whether there was a lasting functional change in the athletic hearts compared to the controls. The two-sample unequal variance Welch t-test was used to determine statistical significance between the two groups, where p<0.05. It was observed that the athletes had increased LVEDV and mass. The LVEDV was 135 mL in athletes and 115 mL in the controls. The LV mass was 179 ± 53 g in athletes and 132 ± 20 g in controls, p<0.05. There was also decreased stiffness in the athletes (187 ± 43 g/s^2^ in athletes vs. 235 ± 31 g/s^2^ in controls, p<0.05) and increased preload in both ventricles (11.8 ± 2.4 cm in athletes vs. 9.3 ± 1.4 cm in controls, p<0.05). There was no significant difference seen in the right side of the heart and there was no significant change in the CO at rest (7.48 ± 1.5 in athletes and 7.7 ± 1.5 in controls, p=0.638).

Pelliccia et al. [15] investigated cardiac remodelling that occurs over a longer period of time and also directly evaluated the reversibility of cardiac remodelling. These researchers also used an echocardiogram to determine LV cavity size and anterior ventricular septal and posterior free wall thickness. An electrocardiogram (ECG) was used to identify hypertrophy by inputting the QRS voltages in the Sokolow-Lyon index equation (SV1+RV5 or RV6 ≥35 mm). Pelliccia et al. [15] recruited 40 male world-class endurance athletes (rowers n=29, canoers n=5, cyclists n=4, and tennis players n=1, and middle-distance runners n=1) and 20 male participants who played recreational sports. Echocardiogram and ECG measurements were recorded 24 hours after the participant’s last competition and they were re-evaluated after 5.6±3.8 years (range=1-13 years) of deconditioning. Statistical significance was determined by a paired Student’s t-test and Wilcoxon test. A two-tailed p-value <0.05 was considered a significant difference within the two groups after deconditioning. The researchers determined that in athletes after deconditioning, the LV size decreased from 61.2±2.9 to 57.2±3.1 mm (p<0.001), the maximum wall thickness decreased from 12.0±1.3 to 10.1±0.8 mm (p<0.001), and LV mass normalized to a height from 194±25 to 140±21 g/m (p<0.001). However, there was significantly persistent LV cavity dilation in nine athletes where increased body weight and continuation of moderate exercise accounted for this result (multiple regression analysis, R^2^=0.45; p<0.001). 

Neilan et al. [16] researched if there was a difference in cardiac remodelling in relation to the number of hours of endurance training completed. They used an echocardiogram to measure changes in chamber function via RV strain. RV strain is defined as the fraction of change in length over the baseline length ([L1-L0]/L0) [17]. The researchers also measured cardiac markers to determine if there was myocyte injury or stress (cTnT and NT-proBNP, respectively). Neilan et al. [16] recruited 60 non-elite runners who would participate in either the 2004 or 2005 Boston Marathon and were stratified according to the hours of training completed before the marathon (group A: ≤35 miles/week, n=17; group B: 36 to 45 miles/week, n=20; group C: >45 miles/week, n=23). Measurements were conducted 24 hours before and after the Boston Marathon. A one-way ANOVA test was used to compare the three groups and if there was a significant difference, a paired Student t-test was completed where p<0.05 would be a significant result. The pre-marathon echocardiographic results were not significantly different between the three groups. After the marathon, group A had increased RV strain compared to group C, -25±4% and -16±5%, respectively. The cardiac markers were also significantly greater in group A versus group C (cTnT 0.09 and <0.01 ng/mL, respectively; NT-proBNP 182 and 106 pg/mL, respectively). In all of these results p<0.001.

Myocardial Fibrosis and Inflammation

La Gerche et al. [10] examined whether there was myocardial fibrosis along with cardiac dysfunction in endurance athletes. The researchers recruited 40 athletes and obtained an echocardiogram, cardiac magnetic resonance imaging (CMRI), and cardiac marker (cTnT and NT-proBNP) data. The electrocardiogram and cardiac markers served the same purpose as in the aforementioned literature. The CMRI utilized delayed gadolinium enhancement (DGE) as a parameter for myocardial fibrosis. Injured myocardium displays delayed washout of contrast agent in comparison to normal myocardium [18]. Therefore, the fibrosed myocardium would continue to show enhancement on CMRI while the normal myocardium would not. The echocardiogram, CMRI, and cardiac marker measurements were recorded at baseline, immediately after an endurance race (3-11 hours duration) and 6-11 days after the race. A one-way ANOVA test was used to compare the three groups and if there was a significant difference, a paired Student t-test was completed (p<0.05 would indicate significant results). Right ventricular ejection fraction (RVEF, %) decreased from 51.0 ± 3.6 pre-race to 50.0 ± 3.8 approximately one week after the race (p<0.0001). In contrast, the LVEF (%) did not have any significant change after the race. There was also a significant increase in RV strain approximately one-week post-race from −27.2 ± 3.4% to −25.6 ± 3.0%, p<0.05. Second, cTnT was undetectable in most of the participants before the race; however, the median cTnT in the nine athletes who had detectable levels at baseline was <0.015 µg/L. The median cTnT significantly increased in all of the participants post-race (0.018 µg/L, p< 0.0001). The post-race BNP was also significantly increased compared to baseline (13.1 ± 14.0 to 25.4 ± 21.4 ng/L, p = 0.003). There was a significant correlation between the decrease in RVEF (%) and both cardiac markers where r = 0.494, P = 0.002 for cTnT and r = 0.518, P = 0.001 for BNP. Finally, the CMRI analysis showed that DGE was present in the runners who have been competing in endurance sports for longer than those who did not show DGE (20 ± 16 vs. 8 ± 6 years, P = 0.043). It was also observed that RVEF (%) was decreased more in athletes with DGE (47.1 ± 5.9 vs. 51.1 ± 3.7%, P = 0.042).

Mohlenkamp et al. [19] studied the association between long-term endurance exercise, coronary artery calcification (CAC), and myocardial fibrosis. The researchers examined 108 apparently healthy male marathon runners who were ≥50 years. All participants competed in at least five marathons within the last three years of the study and have already completed a median of 20 marathons in total. The control group (n=216) was nonmarathon runners who were matched by age and other cardiovascular risk factors such as smoking and diabetes. Mohlenkamp et al. [19], measured all participants’ CAC scores using electron beam computed tomography (EBCT). The presence of DGE with the CMRI was also measured as in the previous studies. The differences between the two groups were evaluated using a paired t-test, where p<0.05 was considered a significant difference. The odds ratios for CAC scores and the number of marathons completed in relation to the presence of DGE were statistically analysed using Spearman correlation and confirmed by logistic regression. It was discovered that there was no difference in CAC scores between the two groups. It was also observed that only 12 participants in total displayed DGE. However, the marathon runners who also displayed DGE had a significantly higher CAC score. The median CAC (Q1-Q3) was 192 (129-603) in the runners and 26 (0-159) in the controls, P=0.0046. The odds ratio of CAC scores and DGE was 1.17 (p<0.05) and the odds ratio of the number of marathons completed and DGE was 1.62 (p<0.05).

Wilson et al. [20] investigated the effects of endurance exercise on cardiac function and the presence of myocardial fibrosis. The researchers recruited 12 male endurance athletes who have competed in endurance competitions throughout their lives (56 ± 6 years old), 20 age-matched non-athletic controls (60 ± 5 years old), and 17 young male endurance athletes (31 ± 5 years old). They used the CMRI to measure cardiac function and used DGE as a measure of fibrosis. A one-way ANOVA test with a post-hoc Bonferroni was used to compare the three groups. A Mann-Whitney test, where p<0.05, was used to compare the older athletes and age-matched controls. The RVEF (%) and LVEF (%) was significantly decreased in the older athletes compared to the controls. RVEF (%) was 66 ± 5 and 72 ± 6 (p<0.05) in the older athletic group and control group, respectively. LVEF (%) was 66 ± 5 and 71 ± 4 (p<0.05) in the older athletic group and control group, respectively. It was also observed that the older athletes had a significantly smaller LVEDV, LV end-systolic volume (LVESV), RV end-diastolic volume (RVEDV), and RV end-systolic volume (RVESV) than the younger athletes (p<0.05). There was no DGE in the control group or young athlete group; however, half of the older athletes displayed DGE - this result was determined to be linked to the number of years spent training (P<0.001) and marathons completed (P<0.001). 

Benito et al. [9] also investigated the association between myocardial fibrosis and endurance exercise. They examined 23 male Wistar rats which were randomly assigned to the exercise group (n=12) or sedentary group (n=11). The exercise group exercised five days a week with 60-minute of running at 60 cm/s. Echocardiographic data on the heart mass were measured at 4, 8, and 16 weeks in both groups. After 16 weeks, the rats were euthanized and messenger RNA (mRNA) of transforming growth factor-β1 (TGF-β1), fibronectin-1, matrix metalloproteinase-2 (MMP-2), tissue inhibitor of metalloproteinase-1 (TIMP1), procollagen-I, and procollagen-III was measured using polymerase chain reaction (PCR) and Western blot analysis. In order to further measure fibrosis, cardiac tissue was examined for collagen and hydroxyproline using Picosirius red stain. A two-way ANOVA was used to evaluate echocardiographic and PCR data while a one-way ANOVA was used for Picosirius red and Masson-trichrome stain results. If there was a significant result, a Bonferroni-corrected t-test was used between the two groups. There was no significant difference in the experimental and control group before eight weeks; however, after 8 and 16 weeks, there was significant LV dysfunction, which was indicated by decreased S wave in pulmonary vein flow and increased LV isovolumic relaxation time (p<0.05). All mRNA levels were significantly increased in the exercise rats after 16 weeks of exercise. Benito et al. [9] also measured the same parameters after two weeks of deconditioning and all of the pro-fibrotic markers returned to normal levels.

Sinus Node Disease and Arrhythmias

Calvo et al. [11] investigated the association between lone atrial fibrillation (LAF) and endurance exercise using a case-control study. LAF is the presence of AF without any underlying heart or systemic conditions. The researchers examined 115 cases of LAF and 57 controls to investigate different risk factors associated with LAF. The significant variables associated with LAF were determined by using conditional logistic regression and backward-stepwise selection. After it was found that endurance exercise had a significant association with LAF, receiver-operating characteristic (ROC), and positive likelihood ratio was used to determine the cut-off points for lifetime hours of intense endurance exercise. The participants were stratified into >2000 hours and <2000 hours of lifetime endurance exercise. The sample sizes were estimated in order to yield 80% power, where p<0.05 would indicate a significant AF-risk odds ratio; these conditions only applied if the prevalence of intense endurance exercise was 50% and 33% in the LAF group and control group, respectively. It was determined that there were increased odds of developing AF for those who completed ≥2000 hours of lifetime endurance exercise compared to sedentary cases (OR 3.88 [1.55-9.73]). On the other hand, <2000 hours of intense endurance exercise significantly protected against AF (OR 0.38 [0.12-0.98]). It was also observed that less than 1000 hours of lifetime intense exercise resulted in increased odds of developing AF.

Baldesberger et al. [21] investigated SND and atrial flutter in former endurance athletes. SND was defined as bradycardia (50 beats/minute in the day-time, 40 beats/minute in the night), maximal RR interval >2.5 seconds, atrial flutter, or pacemaker implantation. The researchers recruited 134 former Swiss professional cyclists (66+7 years old) and 62 male golfers for the control group (66+6 years old). The former cyclist's last race was 38+6 years ago. The 24-hour Holter ECG and echocardiographic data were obtained from all participants. Categorical data were statistically evaluated with Fisher’s exact test and Chi-squared test; meanwhile, the continuous data were analyzed with the Mann-Whitney test, where p<0.05 indicated a significant result. The echocardiographic data showed that the LVEF (%) was decreased in former athletes compared to controls (62+8 vs. 64+6%, p<0.05). In the former athletes, the LV end-diastolic diameter, the LA and RA size indexes, and the LA and RA volume indexes were larger in comparison to the controls (p<0.05). There was no significant difference in the interventricular septum thickness and muscle mass index. The Holter ECG showed that ventricular tachycardia occurred more frequently in the former athletes than the controls (15% and 3% p<0.05, respectively). It was also observed that paroxysmal or persistent atrial fibrillation/flutter and SND occurred more often in former athletes (p<0.05). There was no significant difference in the frequency of isolated atrial/ventricular premature complexes or supraventricular tachycardias.

Guasch et al. [14] explored AF inducibility related to changes in vagal tone after increased endurance exercise. The researchers used 14 Wistar rats in the experimental group and 20 rats in the control group. The experimental group rats were subjected to one hour a day of treadmill running at 28 m/minute for five days a week. The exercise rats completed this exercise regimen for either 8 or 16 weeks. After exercise exposure, the rats underwent an in vivo electrophysiological study where the RA was stimulated by a 1.9-F octapolar catheter. AF was considered present if there was >1 second of irregular atrial and ventricular, non-sustained if AF was 1-30 seconds, sustained if AF was 30 seconds - 15 minutes, and long-lasting if AF occurred for more than 15 minutes. Subsequently, central vagal tone changes were assessed by administering phenylephrine and observing reflex bradycardia. Finally, regulators of G-protein signalling (RGS) and IKACh sensitivity to carbachol were studied since the RGS inhibitory action on IKACh is a potential mechanism for exercised induced end-organ variations in vagal tone. It was observed that there were no significant differences between the two groups at eight weeks of exercise. However, after 16 weeks there was greater inducibility of AF >30 s in the exercise group compared to the sedentary group (64% and 15% p<0.01, respectively). There was also enhanced sensitivity to phenylephrine-induced reflex bradycardia and increased IKACh sensitivity to carbachol with RGS downregulation (p<0.05). The parasympathetic tone was restored after four weeks of deconditioning.

Discussion

Cardiac Dysfunction and Remodelling

The purpose of this literature analysis was to determine whether cardiac dysfunction, myocardial fibrosis, and arrhythmia are inducible by intense endurance exercise. Cardiac dysfunction and remodelling related to endurance training were studied by Apor et al. [6], Pelliccia et al. [15], and Neilan et al. [16]. The results from Apor et al. [6] implied that there is no cardiac dysfunction at rest even though there is evident remodelling present in their athletic participants. These results are mostly explained by the athlete’s heart; thus, there were no symptomatic pathologies or dysfunctions related to the cardiac remodelling in young endurance athletes.

In contrast, Pelliccia et al. [15] showed that there was the presence of RV diastolic dysfunction and myocyte damage caused by endurance exercise; however, less training (less hours of endurance exercise overall) caused more damage to the heart after a marathon than in those who trained longer. This may have occurred because athletes in this study trained enough in order to develop the athlete's heart and were capable of compensating for the increased oxygen demand during the marathon, as seen in Apor et al. [6]. However, those who did not train long enough did not develop the athlete's heart and they exacerbated the limits of their cardiac function during the marathon, which resulted in myocyte damage and dysfunction. 

Finally, Neilan et al. [16] indicated that there was significant RV dysfunction related to remodelling and fibrosis following an endurance race, especially in those who previously participated in very long hours of endurance exercise. Of these three articles, the study by Neilan et al. [16] was the only one that supported myocardial dysfunction and remodelling occur due to intense endurance exercise. This could be explained by the possibility that all participants in this study who practiced very long hours of endurance training, exacerbated the benefits from athlete’s heart seen in Apor et al. [6] and Pelliccia et al. [15]. As well, none of the participants who practiced fewer hours of endurance training were below the lower limit of exercise which would also cause the RV dysfunction seen in the study by Pellicia et al. [15]. Therefore, these articles display that both too little and too much endurance exercise could cause cardiac dysfunction and that cardiac remodelling could be exacerbated by participating in endurance activates without proper training. However, the clinical significance of cardiac dysfunction could not be consistently demonstrated in these studies.

Myocardial Fibrosis and Inflammation

Myocardial fibrosis in relation to endurance exercise was studied by La Gerche et al. [10], Wilson et al. [20], Benito et al. [9], and Mohlenkamp et al. [19]. The study by La Gerche et al. [10] suggested that increased myocardial fibrosis and chamber dilation plays a role in decreased EF one week after an endurance race. The study by Wilson et al. [20] found that an increased number of marathons completed and an increased number of years of endurance training were associated with myocardial fibrosis. Third, Benito et al. [9] clearly displayed the presence of cardiac dysfunction and myocardial fibrosis from endurance exercise via mRNA studies and collagen staining in rats. Finally, Mohlenkamp et al. [19] additionally studied the effects of coronary artery calcification and myocardial fibrosis from endurance exercise. The results from this study found that increased endurance exercise could cause myocardial fibrosis and that myocardial fibrosis increased the odds of developing higher CAC scores. Although these articles used different methods and parameters, they were all consistent with each other and supported that myocardial fibrosis occurs due to intense endurance exercise. However, the clinical significance of myocardial fibrosis in endurance athletes could not be determined from these articles.

Sinus Node Disease and Arrhythmias

The articles by Calvo et al. [11], Baldesberger et al. [21], and Guasch et al. [14] studied the inducibility of SND or arrhythmia from intense endurance exercise. First, Calvo et al. [11] found that there was a potential upper and lower limit of endurance exercise in relation to atrial fibrillation (2000 hours and 1000 hours, respectively). Baldesberger et al. [21] investigated SND and atrial flutter in former endurance athletes and found that there was increased SND and atrial flutter in the former athletes compared to non-athletes. Guasch et al. [14] displayed that there was enhanced AF inducibility and increased central and end-organ vagal tone after 16 weeks of endurance exercise in rats. The results from all three articles were consistent and supported that clinically significant atrial fibrillation and arrhythmias could be increased in those who participate in intense endurance exercise.

Strengths and Limitations

Although the articles in this literature analysis shed light on cardiac dysfunction and remodelling, myocardial fibrosis, and arrhythmia induced by endurance exercise, all of these studies faced many limitations.

Notable strength in the study by Apor et al. [6] was that the researchers examined young endurance athletes at rest using PDF formalisation. This parameter used the echocardiographic data to provide insight into the load-independent factors that influenced diastolic function in otherwise healthy subjects. It also examined whether those with athlete’s heart had any subclinical cardiac dysfunction related to remodelling. A major weakness of this study was that the sample size was too small to draw any clinical conclusions. In addition, Apor et al. [6] were unable to differentiate the effects between strength-dominant and endurance-dominant training since the subjects of this analysis participated in professional canoeing - a combined strength-endurance sport.

The study by Pelliccia et al. [15] was particularly strong in providing an adequate sample size and examining how the amount of training affected remodelling and reversibility. They also investigated other factors that could slow down reversibility; such as, weight gain or continuation of exercise. Some limitations of this study were that all of the athletes were young (mean age 33 years old); therefore, the subjects’ ability to reverse changes may be stronger than those who continue to exercise and decondition at an older age. Pelliccia et al. [15] also noted that they could not exclude the slow development of dilated cardiomyopathy since the echocardiographic data were not sufficient in examining the tissue on a microscopic level. Finally, a major strength in the study by Neilan et al. [16] was that the remodelling and myocyte injury/stress were clearly correlated to the amount of endurance training completed. A limitation of this study was that the long-term implications of endurance exercise were not directly investigated since measurements were gathered shortly after an endurance race. 

Even though the articles that studied myocardial fibrosis were consistent with each other, there were various strengths and weaknesses within each study. The strengths in the study by La Gerche et al. [10] were that the effects of myocardial fibrosis and cardiac remodelling were directly linked to cardiac dysfunction, which shed light on possible mechanisms that endurance exercise affects the heart. However, long-term consequences of endurance exercise were not investigated since measurements were taken immediately after the race and one week after the race. The participants were also considered to be young athletes (mean age: 37 ± 8 years old). Thus, conclusions could not be drawn on the effects on the older population after many years of endurance exercise or deconditioning. Benito et al. [9] also provided strong evidence for a pathophysiological mechanism for the increased myocardial fibrosis observed from endurance training and it also demonstrated the reversibility of these changes. However, the weakness was that the sample size was very small and that clinical implications in humans could not be certain.

The study by Wilson et al. [20] was particularly strong in that fibrosis was assessed in two different age populations and different amounts of endurance exercise completed. However, there were a few limitations to this study. The older athletic sample size was too small to draw clinical conclusions since it was a very unique population. In addition, echocardiographic function tests in relation to fibrosis; such as strain and EF, were not assessed. The researchers only compared morphological changes and fibrosis between the two groups.

Finally, the limitations in the study by Mohlenkamp et al. [19] was that although correlations between CAC and myocardial fibrosis were made in relation to endurance exercise, the cohort size did not have enough power for clinical implications to be drawn. In addition, the other factors affecting CAC could not be fully controlled with complete certainty.

The articles that evaluated the inducibility of arrhythmias and SND from endurance exercise also has some limitations and strengths. The strengths in the study by Calvo et al. [11] were that specific hours of exercise related to LAF were determined using ROC. In addition, other risk factors associated with LAF were investigated along with endurance exercise to determine the combined effect of these risk factors on LAF. Some limitations were that lifetime endurance exercise was determined by a questionnaire which is subject to bias and inaccuracy. Second, CAC was not clearly excluded in the study population, therefore, there could have been some confounding effects. 

The study by Baldesberger et al. [2] provided strong evidence for the direct effect of remodelling on the production of arrhythmia by combining ECG and echocardiographic data. However, there were several limitations to this study. Positive selection bias could have occurred since 24 of the former athletes deceased during the study, the cause of death was not known in 6 of them, and 11 former athletes could not be traced. The use of anabolic steroids and other performance-enhancing drugs (PEDs) was also unknown and could not be assessed in this study. Therefore, future studies could investigate the combined effects of PEDs and endurance exercise on atrial fibrillation.

Finally, the strength in the study by Guasch et al. [14] was the use of mRNA and RGS measurements, which provided strong evidence for a pathophysiological mechanism in AF induced by endurance exercise. The study’s limitations were that the clinical implications in humans could not be made from these animal models. However, the article provides a direction in which the pathophysiological mechanism could be further studied.

Reversibility Related to Remodelling and Fibrosis

Some of the studies in this literature analysis review addressed the reversibility of cardiac remodelling and fibrosis after deconditioning from exercise. Although reversibility in cardiac remodelling was observed in the study by Pelliccia et al. [15], further analysis should be conducted to address if subclinical damage and dysfunction persisted in the older population, despite normalization of chamber size after deconditioning. Benito et al. [9] also observed profibrotic mRNA levels regress to normal levels after deconditioning; however, it should also be determined if the existing fibrosis persists or gets broken down after deconditioning. The reversibility of remodelling and fibrosis is particularly important in the older population because the diagnosis of pathological cardiomyopathies vs exercise-induced heart damage becomes increasingly difficult if it is known that older populations cannot reverse these changes as much as or as quickly as younger populations [22].

*Use of PEDs* 

Guasch et al. [14] addressed the use of PED in some of their participants. It is already known that PED (e.g., anabolic steroids) alone are related to cardiovascular disease (CVD). For instance, an anabolic steroid is associated with increased LV mass and a reduction in diastolic function due to a decrease in myocardial relaxation [23]. PED also allows one to perform at a level beyond their specific biological limits which could induce arrhythmia [24]. Although CVD risk from PED has been thoroughly studied, it has only been assessed as the combined effect of PED and endurance exercise. Further studies should aim to investigate the interaction between the effects of PED and endurance exercise, whether there are synergistic or additive effects on the risk of developing HD. 

## Conclusions

Overall, increased cardiac remodelling, myocardial fibrosis, and arrhythmias were associated with increased endurance exercise in most instances. However, it is not certain whether cardiac remodelling and myocardial fibrosis result in clinically significant cardiac dysfunction after deconditioning. In this literature review, increased incidence of SND and atrial flutter in endurance athletes, even after deconditioning seems to be most associated with endurance exercise. All of the studies reviewed faced a similar limitation of using an adequate sample size since it is difficult to follow and recruit athletes who have an accurate record of lifetime endurance activity. Other variables such as the potential for reversibility and PED use needs to be further investigated. Elucidating the pathophysiological mechanisms in which cardiac changes occur due to endurance exercise could contribute to the exercise recommendations by the CDC and individual recommendations on a physician-patient level.
